# Metabolic Dysregulation in Idiopathic Pulmonary Fibrosis

**DOI:** 10.3390/ijms21165663

**Published:** 2020-08-07

**Authors:** Elena Bargagli, Rosa Metella Refini, Miriana d’Alessandro, Laura Bergantini, Paolo Cameli, Lorenza Vantaggiato, Luca Bini, Claudia Landi

**Affiliations:** 1 Respiratory Diseases and Lung Transplant Unit, Department of Medical and Surgical Sciences and Neurosciences, University of Siena, 53100 Siena, Italy; bargagli2@gmail.com (E.B.); refini@unisi.it (R.M.R.); dalessandro.miriana@gmail.com (M.d.); laurabergantini@gmail.com (L.B.); paolocameli88@gmail.com (P.C.); 2 Functional Proteomics Lab, Department Life Sciences, University of Siena, 53100 Siena, Italy; lorenz.vantaggiato@gmail.com (L.V.); luca.bini@unisi.it (L.B.)

**Keywords:** metabolic dysregulation, idiopathic pulmonary fibrosis, renin–angiotensin–aldosterone system, lipid metabolism, oxidative stress, iron metabolism

## Abstract

Idiopathic pulmonary fibrosis (IPF) is a fibroproliferative disorder limited to the lung. New findings, starting from our proteomics studies on IPF, suggest that systemic involvement with altered molecular mechanisms and metabolic disorder is an underlying cause of fibrosis. The role of metabolic dysregulation in the pathogenesis of IPF has not been extensively studied, despite a recent surge of interest. In particular, our studies on bronchoalveolar lavage fluid have shown that the renin–angiotensin–aldosterone system (RAAS), the hypoxia/oxidative stress response, and changes in iron and lipid metabolism are involved in onset of IPF. These processes appear to interact in an intricate manner and to be related to different fibrosing pathologies not directly linked to the lung environment. The disordered metabolism of carbohydrates, lipids, proteins and hormones has been documented in lung, liver, and kidney fibrosis. Correcting these metabolic alterations may offer a new strategy for treating fibrosis. This paper focuses on the role of metabolic dysregulation in the pathogenesis of IPF and is a continuation of our previous studies, investigating metabolic dysregulation as a new target for fibrosis therapy.

## 1. Introduction

Idiopathic pulmonary fibrosis (IPF) is a severe chronic interstitial lung disease (ILD) of unknown aetiology [1], limited to the lungs. Median survival is from 3 to 5 years after diagnosis [2,3]. The disease is characterized by a radiological and histological pattern of usual interstitial pneumonia (UIP) [4] with parenchymal fibrosis and excess collagen deposition [5]. It mainly affects ex-smoker adults over 65 years, causing dyspnoea, dry cough and the progressive loss of respiratory function [6]. Fibroblast/myofibroblast proliferation and the activation of alveolar epithelial cells with progressive extracellular matrix deposition in the lung parenchyma destroy lung structure [7]. Antifibrotic agents (nintedanib and pirfenidone) reduce the progression of IPF [8,9]. The pathogenesis of IPF remains unclear, despite the growing number of studies. There is an urgent need for reliable biomarkers for early diagnosis and to monitor progression. Recent studies have identified potentially useful peripheral and bronchoalveolar lavage (BAL) biomarkers, including chemokines, cytokines, genes and proteins [10,11,12,13,14,15], but the 2018 International Guidelines for the Clinical Diagnosis of IPF do not include recommendations, due to insufficient evidence [16].

The role of metabolic dysregulation in the pathogenesis of IPF has not been investigated in detail. Several years ago, our research group applied proteomic methods to the bronchoalveolar lavage (BAL) fluid of IPF patients, observing the alteration of certain molecular mechanisms linked to metabolism and fibrosis [17,18,19,20,21]. These alterations concerned the renin–angiotensin–aldosterone system, hypoxia and oxidative stress, endoplasmic reticulum (ER) stress, lipid metabolism and iron metabolism. The involvement of lipid metabolism was an intriguing finding. More recently, we also documented higher concentrations of a peripheral apolipoprotein, serum amyloid A, in IPF patients, than in patients with other interstitial lung diseases. Serum amyloid A proved to be correlated with prognosis and lung function and was proposed as biomarker for this severe fibrotic disease [13]. Lipidomic studies in IPF patients also showed alterations in the synthesis and activity of fatty acids, cholesterol and other lipids [22] that may play a role in cell energy storage, structure and signalling [23,24,25,26,27,28]. Summer et al. commented on a research hypothesis of Chu et al. that IPF dysfunctional alveolar epithelial type II cells become more vulnerable to apoptosis due to stress to the endoplasmic reticulum caused by elevated concentrations of saturated fatty acids. Higher lung concentrations of the saturated fatty acid palmitate were observed in IPF patients than in the controls, suggesting that a link between a palmitate-rich diet and the development of lung fibrosis [29,30]. Chu et al. proposed that a diet rich in fatty acids can induce ER stress and exacerbate experimental pulmonary fibrosis by modulating ER stress [29].

Although IPF is considered to be limited to the lungs and most pathogenetic hypotheses centre on the respiratory system, our results and those of other authors suggest that the disease has systemic elements. We focus on these aspects in the present paper, in particular on the role of metabolic dysregulation in the pathogenesis of IPF.

## 2. Metabolic Alterations in IPF

### 2.1. Proteomic Studies Suggesting Metabolic Alterations in IPF Onset

Proteomic and bioinformatic analysis performed by us on the BAL from IPF patients compared with the BAL of other ILDs, and healthy controls highlighted various dysregulated proteins related to transcriptional factors such as NF-kB, PPAR-γ and c-myc. In particular, the interactomes showed fatty acid binding protein 4 (FABP4), retinol binding protein 4 (RBP4), haptoglobin (HP), apolipoprotein AI (APOAI) and zinc-alpha-2-glycoprotein (ZA2G), related to PPAR-γ; while angiotensinogen (ANGT), surfactant A2 (SP-A), alpha-2-HS-glycoprotein (FETUA), alpha-1-antichymotrypsin (AACT), peroxiredoxin 1 (PRDX1), beta 2 microglobulin (B2M), glutathione S transferase 1 (GSTP1) and transthyretin (TTHY) were related to NF-kB. C-myc was linked to PRDX1, albumin (ALBU) and annexin II (ANXA2) deregulation [17,18]. These three fundamental transcriptional factors are known to be involved in numerous mechanisms related to metabolic disorders [31,32,33,34,35]. Enrichment analysis showed that these factors are correlated with metabolic pathways such as the renin–angiotensin–aldosterone system (RAAS) and hypoxia and oxidative stress [18]. Subsequently, our group worked on a BAL comparison between familial and sporadic IPF. Functional analysis showed that ER stress played a central pathogenic role in familial IPF, confirming that the translation of a genetic variant or dysregulated fatty acids [29] could lead to the engorgement of the endothelial reticulum (ER) [36,37]. On the other hand, proteins up-regulated in the sporadic IPF group were particularly involved in oxidative stress response, as reported previously [19]. In this study, NF-kB and c-myc again played a central regulatory role and the androgen receptor also acted as a transcription factor linked to differential proteins such as ALBU, complement C3, PRDX1 and fibrinogen. Andrisse et al. reported that the androgen receptor, a member of the steroid hormone receptor family, is involved in metabolic disorders [38] and directly binds the regulatory subunit of phosphatidylinositol 3 kinase (PI3K), Foxo1 and CREB promoter regions [38], altering their function. All these processes are known to be related to the onset of fibrosis [39,40,41,42]. In a recent article that compared BAL fluid from stable IPF patients and those subject to acute exacerbations, a central role of macrophages and their activation receptors, liver X receptor (LXR) and farnesoid X receptor (FXR), was found. This activation was particularly evident in IPF patients subject to acute exacerbations [20]. It emerged that the sequestration of modified extracellular lipids by foamy macrophages could be a key event in the evolution of fibrosis towards acute exacerbation [43,44]. The dysregulation of calgranulin A8 (S100A8), A1AT and ALBU suggests “IL-12 signalling and production in macrophages” and the “production of nitric oxide and reactive oxygen species in macrophages”, stressing the role of macrophages in acute exacerbation [20]. Another proteomic study by Landi et al. on the BAL of patients with pulmonary fibrosis related to systemic sclerosis showed the dysregulation of proteins related to lipid metabolism, such as lysozyme C, FABP4, RBP4 and HP, functionally correlated with PPAR-γ, underlining the idea of lipid metabolic alterations concomitant with the onset and development of fibrosis [45].

### 2.2. Metabolic Dysregulation: Renin–Angiotensin–Aldosterone System in Fibrosis

In 2013 and 2014, our proteomic studies on the BAL of IPF patients, compared with the profiles of healthy smoker and non-smoker controls, reported a dysregulation in proteins involved in the RAAS [18]. In particular, the down-regulation of ANGT in the BAL of IPF patients with respect to the other conditions was reported. Angiotensinogen is the substrate of renin and its down-regulation in IPF strongly suggests high renin and ANGT consumption to produce angiotensin I. Indeed, renin converts ANGT into angiotensin I (Ang I), which is in turn cleaved into angiotensin II (Ang II) by angiotensin-converting-enzyme (ACE), also typically found in the endothelial cells of the lung capillaries [46], where RAAS, essential for blood pressure control and fluid homeostasis, plays an important role in pulmonary hypertension. Plasma levels of ANGT are reported to be modulated by hormones such as corticosteroid, oestrogen, thyroid hormones and angiotensin II [46,47], suggesting that hormone imbalance could influence RAAS and its dysregulation. The important role of RAAS in metabolic regulation is also explained by its two axes: ACE-2/Ang 1–7/Mas which has antiproliferative, antioxidant and anti-inflammatory functions with beneficial effects on hypertension, glucose intolerance and insulin resistance (IR); and the counter-regulatory ACE/Ang II/AT1 [48] with opposite effects. ACE-2 has been shown to play a protective role in lung disease through effects mediated by the Mas oncogene. In the lung of IPF patients, ACE-2 is reported to be significantly depleted [49], suggesting that RAAS is important in the pathogenesis of IPF. In turn, the dysregulation of RAAS components can trigger many metabolic functions, such as those observed in metabolic syndrome and diabetes mellitus type 2 [48]. Indeed, in diabetic retinopathy, RAAS is an effector of vascular epithelial growth factor (VEGF) that drive the leakage of retinal blood vessels [50]. VEGF is also well known to be one of the major players in IPF onset and its receptor is one of the targets of nintedanib treatment [11,51].

**Figure 1:**
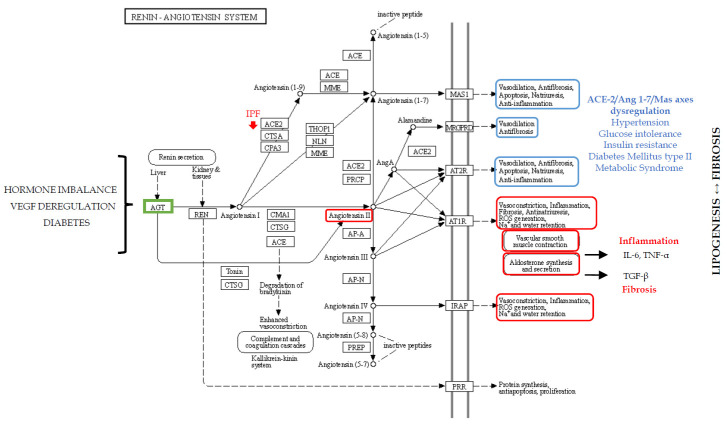
Schematic representation of the renin–angiotensin–aldosterone system (RAAS). AGT (ANGT) in green represent the dysregulated protein that we found with the proteomic analysis. In red and blue, the opposite effects of RAAS.

Moreover, the production of ANGT and Ang II observed in white adipocyte tissue reveals the role of RAAS in promoting adipocyte hypertrophy and decreasing the number of small insulin-sensitive adipocytes [52]. These dysregulations, observed in metabolic disorders, also characterize aging, leading to pro-inflammatory and pro-fibrotic effects in different organs and inducing lung fibrosis, vascular dysfunction, myocardial fibrosis, nephropathy and insulin secretory defects with increased insulin resistance [53]. Angiotensin II is a versatile effector molecule with intracrine/autocrine/paracrine roles. It stimulates the adrenal cortex to secrete aldosterone [54], a pivotal hormone that induces inflammation by the mRNA expression of IL-6 and TNF-α, and fibrosis by TGF-β. It promotes lipogenesis, thus increasing adipose tissue mass [46] and influencing the release of prostaglandins such as cyclooxygenase (COX) 1-derived prostaglandin E(2) [46], found to be defective in epithelial cells and macrophages in the lungs of IPF patients [55]. Cyclooxygenase is required for the production of lipid mediators such as prostanoid (e.g., prostaglandin PGE2) associated with fibrotic lung disorders [56]. Moreover, COX-derived prostanoids appear to play an important role in Toll-like receptor response [56]. Curiously, high glucose in cell cultures induces an increase in angiotensinogen, angiotensin-converting enzyme (ACE) and AT1R mRNA levels, as well as Ang II and TGF-β1 concentrations, promoting the epithelial–mesenchymal transition and fibronectin synthesis [57]. Aldosterone is also reported to impair metabolism and to stimulate the differentiation of 3T3-L1 cells and brown preadipocytes into mature adipocytes, influencing the expression of the adipokine gene [58]. Alterations in RAAS are therefore closely related to metabolic disorders such as diabetes and dyslipidaemia, which may influence fibrosis pathways in different ways (Table 1; Figure 1).

### 2.3. Hypoxia, Oxidative Stress and Iron Metabolism

Our proteomic studies found dysregulated proteins such as HPT, transferrin, PRDX1, PRDX5, GSTP1, CERU and ALBU in the BAL of IPF patients; these proteins are related to hypoxia, oxidative stress and iron metabolism [18,19]. The renin–angiotensin–aldosterone system that we have already described is also known to increase oxidative stress [59], since Ang II is an inducer of reactive oxygen species [59], which together with reactive nitrogen species are reported to be elevated in IPF [45,97,98]. A hypoxic stimulus, characterizing this pathology, increases the activation of hypoxia inducible factor-1α (HIF-1 α), a transcription factor [60], which together with constitutively expressed HIF-1β, determines the transcription of hypoxia response elements (HREs) with pivotal roles in metabolic reprogramming [61]. The transcription of hypoxia response elements triggers adaptive mechanisms, modifying glycolysis, angiogenesis, pro-survival signalling, cell proliferation and cell migration [62]. The HIF-1 target genes include pyruvate dehydrogenase kinase 1, hexokinase 2 and lactate dehydrogenase, the modified expression of which gives rise to a glycolytic switch, similar to the Warburg effect in cancer [62], which also occurs in IPF, as already reported [63]. The Warburg effect is accompanied by high lactic acid production which induces the epithelial–mesenchymal transition via the activation of TGF-β. Moreover, the glycolytic switch to aerobic glycolysis not only produces little energy but also stabilizes HIF-1α, which promotes TGF-β-induced myofibroblast differentiation [18,60,63,64]. Oxidative stress, the induction of HIF-1α and the glycolysis switch are also related to extracellular matrix deposition, typical of fibrosis. Extracellular matrix production is strongly linked to glycolysis, since the latter provides energy and building blocks for collagen production. Another hypoxia response element is lysyl oxidase transcription, fundamental for collagen crosslinking, and typical of fibrotic tissue [64]. In line with the oxidative stress response and extracellular matrix deposition, Stock et al. found that the *SOD2* mRNA expression was markedly suppressed in both systemic sclerosis-ILD and IPF fibroblasts, compared with non-ILD controls, leading to an increasing trend in *ACTA2* mRNA expression and cell proliferation [65].

The hypoxic stimulus characterizing IPF also leads to pulmonary hypertension (PH) by progressive vasoconstriction and vascular remodelling. Indeed, hypoxia induces the proliferation and differentiation of pulmonary artery adventitial fibroblasts into myofibroblasts that ultimately, stimulate the recruitment of inflammatory cells and the release of key regulators. The proliferation and differentiation of pulmonary artery adventitial fibroblasts during chronic hypoxia are mainly dependent on the signal transduction of the p38 pathway and its downstream mediator, HIF-1 [66].

Studies on mesenchymal stem/stromal cells, also used in IPF therapy [67], have shown that HIF-1α expression not only influences glycolytic activity, but also mitochondrial oxidative phosphorylation under hypoxic conditions [61]. The latter normally enables the production of ATP and is a major source of reactive oxygen species. In IPF, alveolar macrophages and mitochondrial reactive oxygen species are reported to be significantly more abundant and associated with a lower expression of mitochondrial-encoded oxidative phosphorylation genes [68].

**Figure 2:**
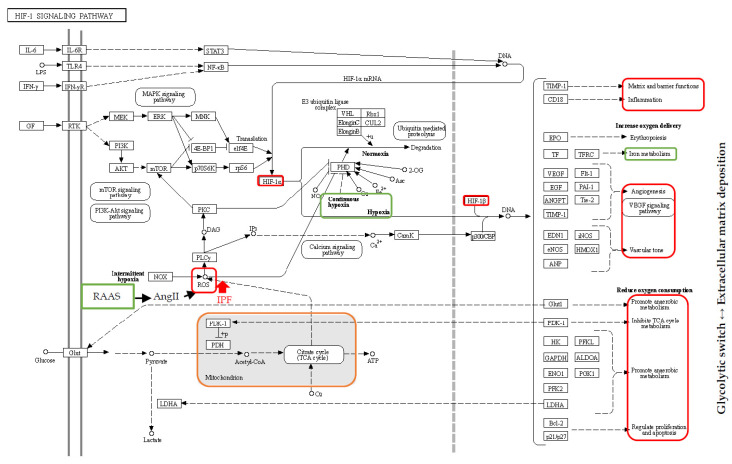
Schematic representation of the HIF1 signalling pathway. In green are highlighted the pathway maps identified by the proteomics and bioinformatics analyses. In red, the modulation and the effects in IPF. In orange, the involvement of the mitochondrial activity.

Another possible source of reactive oxygen species in IPF is the accumulation of iron in the lower respiratory tract, also reported to be induced by smoking. Disordered iron regulation, as our findings suggested, may play a central role in the pathogenesis of oxidative stress-induced microscopic injury, triggering epithelial cell damage and fibroblast proliferation [69]. Patients with IPF typically show excessive extracellular iron and macrophage hemosiderin, suggesting abnormal iron homeostasis leading to recurring microscopic injury and fibrosing damage [69]. Various histological and BAL IPF studies have also shown increased numbers of iron-laden macrophage clusters in the alveolar and interstitial spaces, driving macrophages to generate reactive oxygen species and leading to oxidative damage with DNA breaks, lipid peroxidation and protein oxidation [70]. These findings suggest that oxidative stress and iron metabolic disorder create positive feedback, promoting the progression of fibrosis (Table 1; Figure 2).

## 3. Onset of Fibrosis: The Contribution of Dysregulated Lipid Metabolism

An intriguing interactome that we observed in our work on IPF concerned FABP4, RBP4, HP, APOAI and ZA2G, related to peroxisome proliferator-activated receptor gamma [18]. This intriguing network also emerged in our study, comparing the proteomes of the BAL of patients with pulmonary fibrosis associated with systemic sclerosis and healthy controls [45], corroborating the finding that this pleiotropic nuclear hormone receptor is involved in pathologies that progress to fibrosis. We found FABP4, also known as aP2, an adipokine primarily expressed in adipocytes and in macrophages, in BAL. This adipokine plays a pivotal role in coordinating and integrating metabolic and inflammatory signalling in settings of insulin resistance, dysregulated lipid metabolism and inflammation [71]. FABP4 together with HNF4 and PPAR-α are required to resist oxidative stress and regulate transcription to drive changes in lipid metabolism [72].

RBP4, another protein that we found dysregulated in BAL samples, is a cytokine primarily produced by adipose tissue and connected to PPAR-γ. It is the main transport protein for retinol in the blood. Thus, RBP4 is known to regulate lipids, glucose metabolism and insulin resistance [73]. Retinol bound to RBP4 and STRA6, stimulated by retinoic acid (6), triggers the signalling cascade mediated by JAK/STAT (Janus kinase/signal transducer and activator of transcription) that induces genes that inhibit insulin signalling and control lipid homeostasis [74]. The JAK/STAT pathway is crucial for transducing signals from a variety of metabolically important hormones and cytokines, including growth hormone, leptin, erythropoietin, IL4, IL6 and IFN-ϒ. This pathway is dysregulated in obesity and metabolic diseases [75]. A recent study reported the use of JAK inhibitors on pathologies with lung fibrosis involvement showing that JAK/STAT plays a role in the development of fibrosis [76]. Moreover, other studies highlight lung fibrotic stability after the administration of JAK inhibitors such as Baricitinib in patients with rheumatoid arthritis and lung involvement [77].

Transcriptional factor PPAR-γ, related to our differential proteins, is the master regulator of adipogenesis and has antifibrotic effects initiated by blocking TGF-β in organs such as the skin, lung and heart [78]. In adipogenesis, it determines an increase in mRNA for lipogenic enzymes, adiponectin, insulin-dependent glucose transporter (GLUT4) and ANGT, with effects on lipid and glucose metabolism. Curiously, it has been reported that the first-line antidiabetic drug metformin exerts potent antifibrotic effects by modulating metabolic pathways, activating PPAR-γ signalling, inhibiting TGF-β, suppressing collagen formation and inducing lipogenic differentiation in lung fibroblasts derived from IPF patients [79], but unfortunately it does not have clinically relevant outcomes in patients [80]. Moreover, nintedanib and pirfenidone are reported to inhibit TGF-β in different ways, but do not induce lipogenesis as much as does metformin. Fibroblast growth factor 1 (FGF1) is also linked to PPAR-γ: Mackenzie et al. reported it to be the growth factor most significantly increased in IPF patients, and that it co-localized in basal cell sheets, myofibroblast foci and surfactant protein-C positive alveolar epithelial type-II cells, and with FGFR1, FGFR2, FGFR3, FGFR4 and myofibroblasts [81]. Its relationship to energy intake has earned this growth factor the definition of metabolic regulator. Different environmental signals (exposome) are reported to be sensed by PPAR-γ, which drives adipose remodelling via FGF1. Inadequate levels of FGF1 are also associated with insulin resistance [82].

The action of PPAR-γ is likewise seen in lipofibroblast differentiation, where it prevents oxidative lung injury, promoting mesodermal differentiation and antioxidant defences. PPAR-γ is also reported to indirectly stimulate lung surfactant production, so that the lung can adapt to atmospheric oxygen [83]. PPAR-γ agonists promote adipocyte differentiation and adiponectin secretion [84,85]. Another recent proteomic study of ours on BAL protein modulation after the nintedanib treatment of IPF patients highlighted the up-regulated apolipoprotein C3 in serum after one year of nintedanib administration, linked to PPAR-γ by enrichment analysis [51].

**Figure 3:**
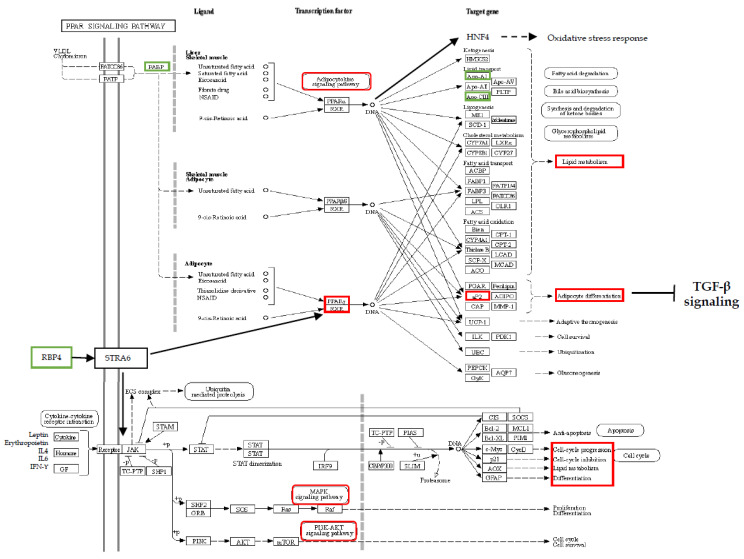
Schematic representation of the PPAR and JAK-STAT signalling pathways. In green are highlighted the proteins identified by the proteomics and bioinformatics analyses. In red, the modulation and the effects in IPF.

An even greater number of studies highlight the lipid metabolic dysregulation in the onset and development of IPF, focusing on PPAR-γ action and the production of adiponectins and leptins [86]. Serum concentrations of adiponectin, as reported by d’Alessandro et al., could be useful for predicting IPF prognosis, since they are inversely correlated with DLco percentages and body mass index. Moreover, adiponectin levels in BAL also demonstrated an inverse correlation with body mass index and a direct correlation with eosinophil percentages, both negative prognostic factors of IPF [86]. Adiponectins are almost exclusively synthesized by adipocytes and are the most abundant adipose tissue-derived adipokine in plasma. They are a powerful hormone involved in lipid metabolism, insulin sensitization, apoptosis and inflammation, besides being a key molecule in several immunological pathways linked to carcinogenesis [87]. Leptin levels are reported to be elevated in serum and correlated with the severity of lung fibrosis, since leptin significantly promotes the epithelial–mesenchymal transition in A549 cells, decreases autophagosome formation, inhibits the lipidation of LC3I to LC3II, and up-regulates the expression of p62 by activating the PI3K/Akt/mTOR pathway [88] involved in the onset and development of IPF (Figure 3).

Interestingly, in line with our findings regarding LXR/RXR and FXR/RXR and the differentiation and activation of macrophages in IPF [20], Venosa et al. reported that treatment with fibrotic substances induces a time-related increase in large vacuolated macrophages and the accumulation of oxidized phospholipids in lung macrophages and epithelial cells, promoting the formation of foamy macrophages and their M2 activation. This treatment also increases phospholipids and cholesterol in BAL fluid, with evident alteration of lipid-handling pathways under the control of the transcription factors LXR, FXR, PPAR-γ and sterol regulatory element-binding protein [89].

### Mitochondrial Alterations at Onset of Fibrosis

Lipid metabolism depends on proper mitochondrial function and correct interaction between mitochondria and ER. Correct outer mitochondrial membrane fusion is therefore essential to connect the intracellular network in order to regulate and meet cell metabolic demands. In turn, mitochondrial fusion is required for oxidative phosphorylation, mitochondrial DNA biogenesis, mitophagy regulation and metabolic adaptation. Chung et al. reported that mitochondrial fusion and lipid metabolism are closely linked to regulate alveolar epithelial cell II injury and subsequent fibrotic remodelling by damaging surfactant lipid production. Surfactants are a lung surface-active lipoprotein complex, containing about 90% lipids, that reduces lung surface tension, prevents the alveoli from collapsing, and plays a critical role in immune regulation [90]. Mitochondria are fundamentally involved in the synthesis of fatty acids, which may be influenced by episodes of insulin-resistance, glycogenesis or the accumulation of glucose. These alterations lead to mitochondrial dysregulation that plays a role in defective autophagy, telomere attrition, altered proteostasis and cell senescence through the increased production of reactive oxygen species, decreased mitochondrial biogenesis and impaired mitochondrial macroautophagy [91,92]. Caporarello et al. reported that restoring PPAR-γ co-activator 1-alpha levels in IPF fibroblasts improved mitochondrial biogenesis and function and NAD biosynthesis, and also influenced the fate of lipogenic fibroblasts through the induction of PPAR-γ transcription [93].

## 4. Discussion

**Figure 4:**
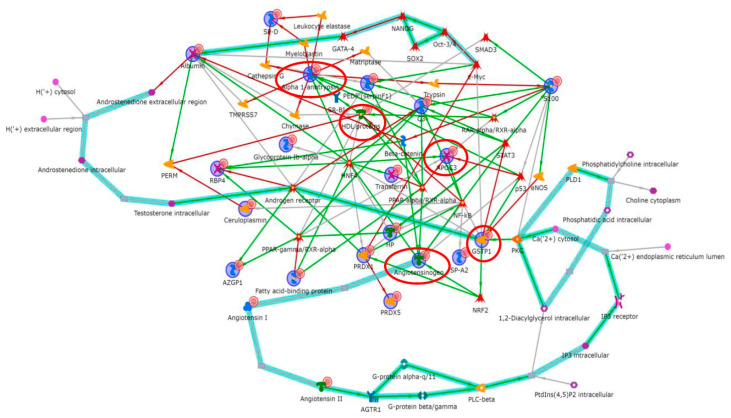
Protein interactome by MetaCore software correlating all the proteins reported in Table 1 in a hub-centric network. APOA1 in HDL, GSTP1, Alpha 1-antitrypsin, Angiotensinogen, APOC3 (red circles) are the central functional hubs i.e., the proteins with a high number of interactions with other modulators in the interactome. The red arrows indicate the inhibition, the green arrows mean inductions, and the grey arrows indicate a generic correlation. The teal highlighted lines indicate the well-known canonical molecular pathways. The relative figure legend is in Supplemental Material in Figure S1.

Proteomics coupled with systems biology studies, performed on BAL samples from patients with IPF, healthy controls, other ILDs and different phenotypes of IPF, has brought to light a number of interesting molecules that seem to be involved in the onset of fibrosis via mechanisms related to metabolic dysfunction [17,18,19,20,45]. We summarize our reports in Table 1, and the proteins indicated and discussed above have been uploaded on MetaCore software™ version 6.37 from Clarivate Analytics (Clarivate Analytics, Boston, MA, USA; https://portal.genego.com/) in order to build the interactome reported in Figure 4. where APOA1, GSTP1, A1AT, ANGT, APOC3 (red circles) are the central functional hubs i.e., proteins with a high number of interactions with other modulators. An --omics approach to the study of IPF has made it possible to obtain a picture of an almost complete set of dysregulated proteins. Bioinformatic analyses linked these proteins to precise molecular pathways, which until recently were tagged as related to specific fibrosis pathways, losing sight of their metabolic effects, and vice versa for the effect that metabolism could have on them. Since recent discoveries regarding the pathogenesis of fibrosis have led to exciting therapeutic opportunities in the field of metabolic regulation, there has been a switch to research on metabolic pathways in IPF.

**Figure 5:**
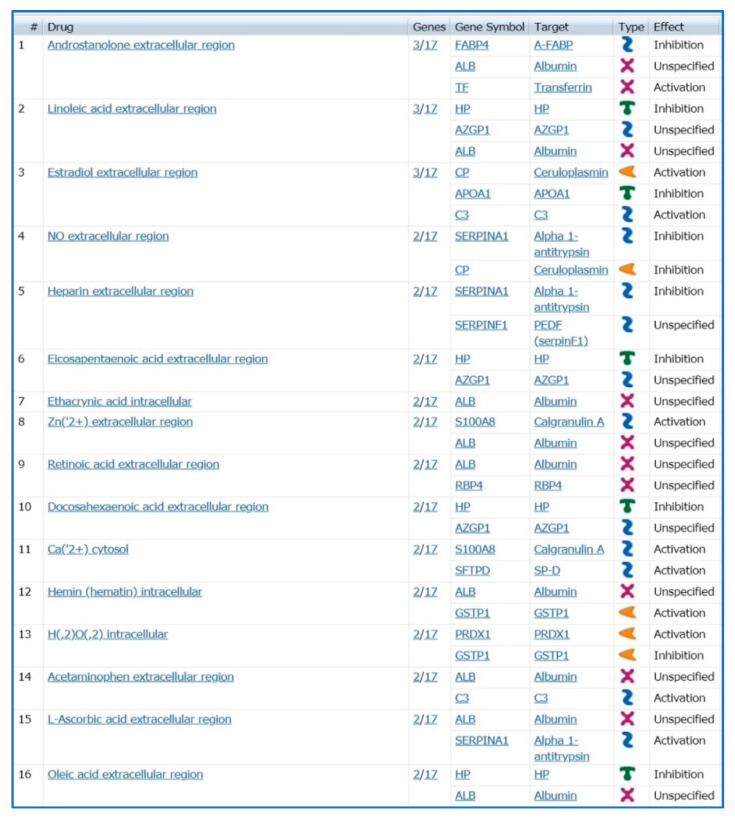
Enrichment analysis performed by the MetaCore software tool “Drug look up for your data”. Figure shows drugs related to the differential proteins reported in Table 1 considered a potential target of treatment.

Following this suggestion, we also performed a bioinformatic analysis on MetaCore software by the tool “Drug look up for your data” visualizing specific drug molecules (Figure 5) related to the differential proteins reported in Table 1, and considered a potential target of treatment. Most of the reported drugs are molecules acting on metabolic and hormonal pathways such as androstanolone, linoleic acid, estradiol, nitric oxide (NO), etc.

Based on our own as well as other findings, a fine balance between lipid metabolism and wound healing mechanisms leading to fibrosis is suggested in IPF onset and development. Lipid metabolism as well as glucose anabolism and catabolism are dramatically subject to certain environmental and genetic influences, which may alter their equilibrium, leading to metabolic or fibrotic disorders. To corroborate these observations, various widespread diseases associated with the onset of fibrosis, including IPF, cirrhosis, hepatitis, non-alcoholic steatohepatitis, chronic kidney disease, myocardial infarction, heart failure, diabetes and scleroderma, share common metabolic pathways [49,57,94,95,96]. The liver and lung are reported to share many immune/inflammatory responses to damage through the lung–liver axis. Metabolic dysregulation described in the liver could have repercussions at the lung level, just as the hepatitis C virus (HCV), which affects the liver, enhances the development of IPF and is considered a risk factor for IPF, and lungs and liver have resident macrophages that play key roles in mediating the immune/inflammatory response [49]. Diabetes is another possible risk factor for the development of IPF [80]. Glycogenesis, the accumulation of glucose and episodes of insulin-resistance also influence the synthesis of fatty acids, leading to mitochondrial dysregulation and contributing to defective autophagy, telomere attrition, altered proteostasis and cell senescence, with an increased production of reactive oxygen species, decreased mitochondrial biogenesis and impaired mitochondrial macroautophagy [91,92]. Interestingly, higher serum and BAL fluid concentrations of hypomethylated CpG-rich mitochondrial DNA, released by necrotic cells and viable cells in response to various stressors, have been reported in IPF patients than in healthy controls. This gives rise to the activation of macrophages and fibroblasts in certain experimental settings, inducing lung fibrosis [91].

Mitochondrial dysregulation is linked to lipid accumulation in the lungs, influencing the lipid composition of pulmonary surfactant, which is reported to be an extracellular lipid reservoir, uniquely susceptible to direct and continuous exposure to environmental oxidants, inflammatory agents and pathogens. Surfactant lipids have been recognized as bioactive molecules that maintain immune quiescence in the lung but can be remodelled by the inhaled environment into neo-lipids, which mediate key roles in inflammation, immunity and fibrosis. Since surfactant composition is sensitive to circulating lipoproteins, the lipid milieu of the alveoli should be recognized as susceptible to diet and common systemic metabolic disorders [99].

## 5. Future Perspectives

Idiopathic pulmonary fibrosis has long been considered to be limited to the lung compartment, although different pathways related to metabolic alterations in IPF are also typical of diseases affecting other organs. Fibrosis is thought to begin in the lung as microvascular injury, leading to endothelial cell damage and alveolar epithelial injury, accompanied by the release of cytokines, chemokines and growth factors, and the induction of the coagulation cascade, triggering positive feedback that amplifies all these molecular responses [100]. Hypertriglyceridemia and hyperglycaemia are widely reported to impair macro- and micro-vascular functions. The inadequate accumulation of lipid depot in the lung after particular kinds of environmental exposure could alter metabolic pathways in this organ. Genetic alterations in different IPF phenotypes could promote these metabolic changes. Variants in different surfactant genes could alter lipid metabolism, while telomere attrition or shortening could influence mitochondrial functions and together they could influence each other to induce a fibrotic response instead of adipose tissue accumulation. Complex diseases such as IPF involve genetic and environmental risk factors and engage many cells and tissues. For this reason, they pose challenges to traditional approaches that examine individual factors in favour of multi-tissue multi -omics systems biology, to comprehensively elucidate the within- and cross-tissue molecular networks underlying gene-by-environment interactions. By combining different -omics data, bioinformatic instruments can be used to correlate the larger amount of information in a network-based systems medicine.

## 6. Conclusions

All the metabolic pathways described are implicated in the pathogenesis of IPF through the induction of the onset and development of fibrosis. Little data are available on the IPF of metabolic pathways, leading to alterations in lipid and glucose metabolism and mitochondrial dysfunctions. Research is increasingly focusing on this new view of IPF as a systemic disorder. The altered metabolism of carbohydrates, lipids, proteins and hormones has been documented in the lung, liver and kidney fibrosis. Correcting these metabolic alterations is becoming a new strategy for antifibrotic therapies. Understanding the pathology of IPF from different but interconnected points of view, namely the exposome, genetic predisposition and dysregulated metabolic pathways, could be the key to a holistic picture of this disease.

## Figures and Tables

**Table 1:** Table summarizing the metabolic dysregulation in idiopathic pulmonary fibrosis (IPF) suggested by different proteomic studies reported in the text, differentially abundant proteins found for each dysregulation, influencing or influenced mechanisms and diseases or effects induced by the relative dysregulations.

Metabolic Pathways Dysregulation in IPF	Dysregulated Proteins Found by Proteomics	Influencing or Influenced Mechanisms	Diseases and Effects	References
Renin-angiotensin-aldosterone system (RAAS)	ANGT	ACE-2/Ang 1-7/Mas axis vs. ACE/Ang II/AT1-R Adipocyte modulation Small insulin-sensitive adipocytes modulation AngII has intracrine/autocrine/paracrine roles Secretion of aldosteron Release of cyclooxygenase (COX) 1-derived prostaglandin E(2) Hormone imbalance	Metabolic syndrome Diabetes mellitus type 2 Lung fibrosis Vascular dysfunction Myocardial fibrosis Nephropathy Hypertension Inflammation by IL-6 and TNF-α Fibrosis by TGF-β.	[46,47,48,49] [50,51,52,53] [54,55,56,57,58]
Hypoxia, oxidative stress and iron metabolism	HPT, TRFE, PRDX1 PRDX5, GSTP1, CERU, ALBU	RAAS increase oxidative stress HIF-1α and HIF-1β determine transcription of HREs HREs modify glycolysis HREs modify angiogenesis HREs modify pro-survival signalling, cell proliferation and cell migration HIF-1α promotes TGF-β-induced myofibroblast differentiation Extra Cellular Matrix (ECM) deposition Deregulation of mitochondrial oxidative phosphorylation (OXPHOS) Excessive extracellular iron and macrophage haemosiderin	Aging Warburg effect Recurring microscopic injury and fibrosing damage Worse fibrosis evolution	[59,60] [61,62,63,64,65] [66,67,68,69,70]
Lipid metabolism dysregulation	FABP4, RBP4, HP, APOAI, ZA2G, APOC3, S100A8 TRFE, C3, PEDF, A1AT A1BG, ALBU, SFPA2, SFTPD	PPAR- ϒ modulation FXR modulation LXR modulation Modulation of JAK/STAT pathway Modulation of adipogenesis and fibrosis by TGF-β	Obesity Metabolic diseases Skin, lung and heart fibrosis Changes in lipid metabolism Regulation of glucose metabolism and insulin resistance	[71,72,73] [74,75,76,77,78] [79,80,81,82,83] [84,85,86,87,88] [20,51,89,90,91,92]
Mitochondrial alterations	FABP4, RBP4, HP, APOAI, ZA2G, APOC3, S100A8 TRFE, C3, PEDF, A1AT A1BG, ALBU, SFPA2, SFTPD	Oxidative phosphorylation Mitochondrial DNA biogenesis Mitophagy regulation Metabolic adaptation Synthesis of fatty acid	Insulin-resistance Defective autophagy Telomere attrition Altered proteostasis Cell senescence Increased production of reactive oxygen species	[93,94,95,96]

## Supplementary Materials

The following are available online at https://www.mdpi.com/1422-0067/21/16/5663/s1, Figure S1: Legend of MetaCore interactome.
