# Supplementary figures and images for: Metabolic Dysregulation in Idiopathic Pulmonary Fibrosis
Source: Int J Mol Sci. 2020 Aug 7;21(16):5663. doi: 10.3390/ijms21165663 (PMC7461042; doi:10.3390/ijms21165663)

## Slide 1
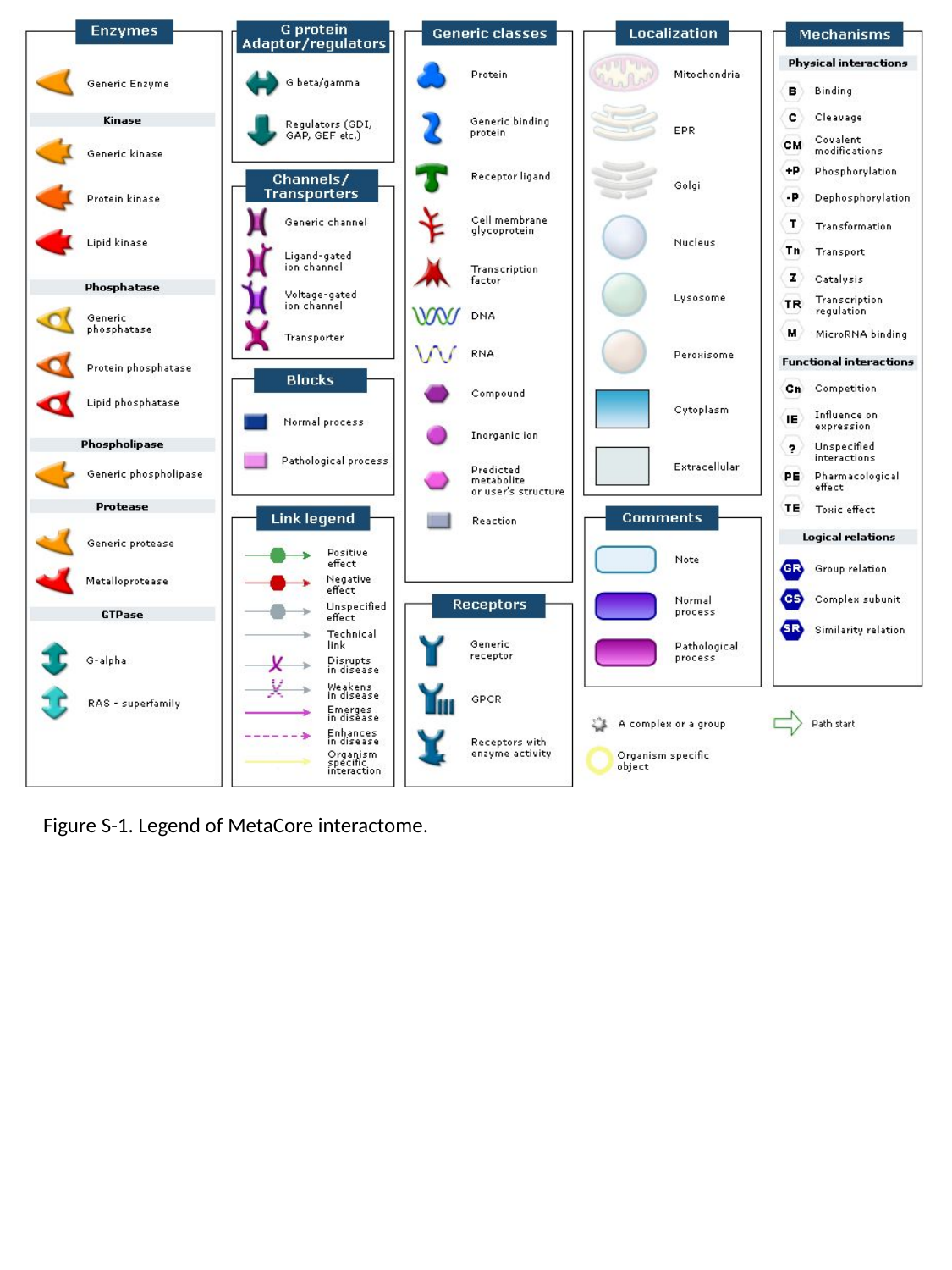

Figure S-1. Legend of MetaCore interactome.

Supplement: Supplementary file 1 [file ijms-21-05663-s001.pptx]
